# CASE REPORT Upper-Limb Disarticulation for Life-Threatening High-Flow Arteriovenous Malformation: Report of a Case

**Published:** 2010-12-22

**Authors:** Yi-Hsuan Su, Vasu Karri, Lai-Fan Lu, Hung-Chi Chen

**Affiliations:** Department of Plastic & Reconstructive Surgery, E-Da Hospital/I-Shou University, No 1 Yi-Da Road, Jiau-Shu Village, Yan-Chao Township, Kaohsiung County, Taiwan, Republic of China

Arteriovenous malformations (AVM) are high-flow lesions having a direct connection between an artery and vein, with bypass of the capillary bed.^1^ Those affecting the upper limb and causing cardiac decompensation are rare.

Treatment of such lesions remains a surgical challenge. Despite multiple procedures, total eradication is often not achieved. For some patients, limb amputation becomes necessary and carries significant risk.

We report a case of congenital AVM of the upper limb causing cardiac decompensation. The patient was safely and successfully treated by performing a 2-stage upper-limb amputation.

## CASE REPORT

**Figure 1:**
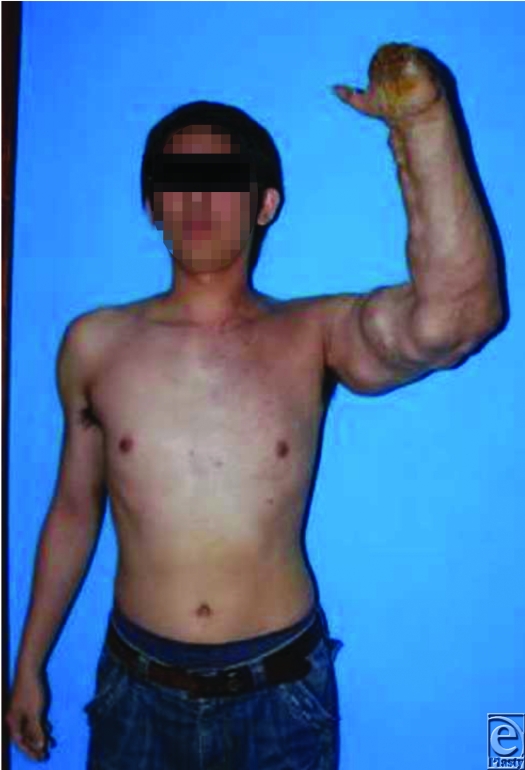
High-flow arteriovenous malformation affecting the entire left upper limb.

A 32-year-old man presented with a congenital high-flow AVM involving the left upper limb and anterior chest (Fig 1). In recent years, the AVM had undergone rapid expansion causing the patient significant pain, high-output cardiac failure, and dyspnea on minimal exertion. The pain was resolved by elevation of the arm.

In addition, there was a chronically infected wound on the dorsum of the left hand from which he suffered a massive bleed 6 years previously. On that occasion, he lost 3500 mL of blood. Amputation of the digits through the metacarpophalangeal joints had been previously performed at another hospital. At presentation, the left upper limb was warm and hypertrophic with pulsating vessels. Bruits were audible and a thrill palpable over the axillary artery. A dry eschar covered the chronic wound on the dorsum of the hand. The vascular malformation over the anterior chest manifested as an uncomplicated reddened patch.

**Figure 2:**
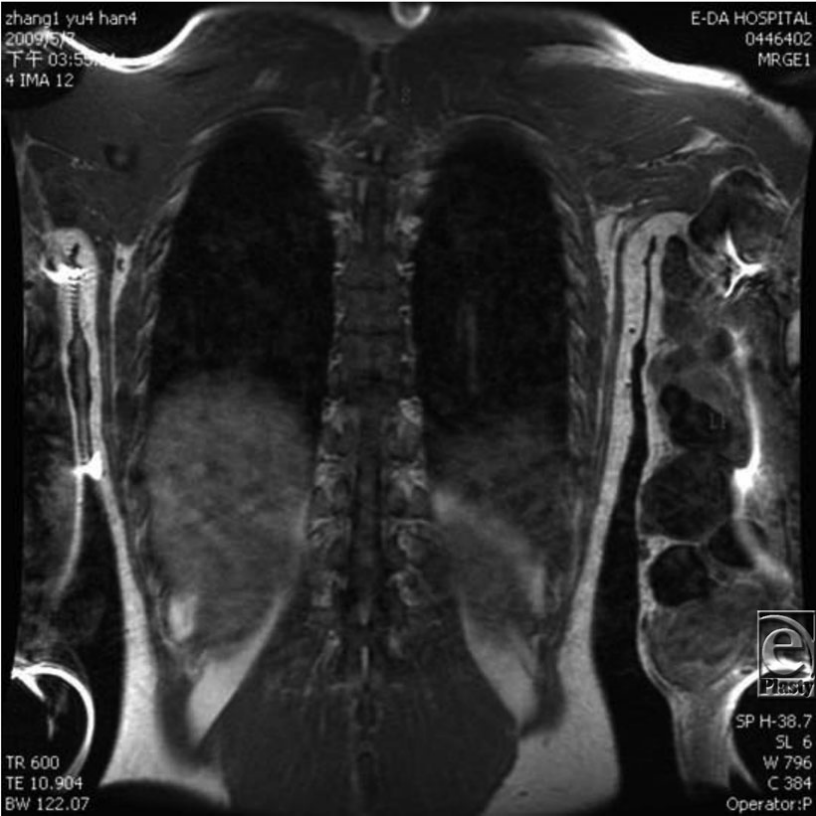
Magnetic resonance imaging revealed an extensive, diffuse arteriovenous malformation involving all compartments of the upper limb. There was no skeletal hypertrophy.

Magnetic resonance imaging (MRI) was performed to determine extent of involved tissues. This revealed an extensive diffuse AVM involving all compartments of the left upper limb. There was no skeletal hypertrophy (Fig 2). Selective embolization was discounted for a number of reasons: the limb was nonfunctional; there was a risk of life-threatening hemorrhage from the chronic wound; embolization would not obliterate the multiple nidi; and partial excision following embolization would carry the risk of uncontrollable intraoperative bleeding, postoperative complications, and recurrence of the AVM. Hence, it was decided at the outset to perform amputation of the affected upper limb. To minimize the risk of uncontrollable intraoperative bleeding and wound-healing problems, amputation in 2 stages was planned.

The first stage comprised disarticulation through the elbow. We intentionally avoided amputation through bone to avoid the risk of uncontrollable bleeding from the bone end. To minimize intraoperative bleeding, a tourniquet was inflated above the elbow and 1/0 PDS (Polydioxanone, Ethicon) transcutaneous sutures placed proximal to the incision. The temporary sutures served to compress cutaneous and subcutaneous vessels thereby reducing bleeding. Consequently, it became easier to formally transfix these vessels during the amputation.

**Figure 3:**
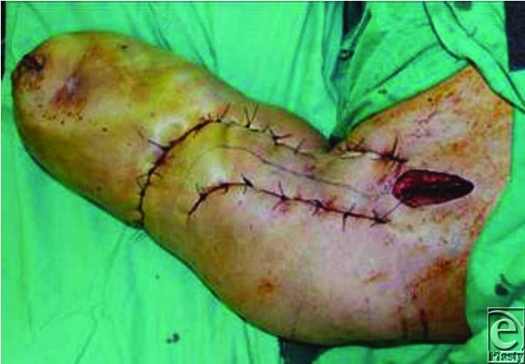
Ligation of the subclavian vessels (open wound) and placement of 2 rows of transcutaneous 1/0 PDS sutures. Skin was incised between these 2 rows of sutures.

The second stage was performed 1 week later. Cardiovascular surgeons performed ligation of left subclavian vessels (Fig 3), immediately followed by disarticulation through the glenohumeral joint. Temporary transcutaneous sutures were used in a similar manner as in the first stage. Multiple, dilated vessels were encountered and ligated or transfixed. Some of the vessels matched diameter of the aorta.

**Figure 4:**
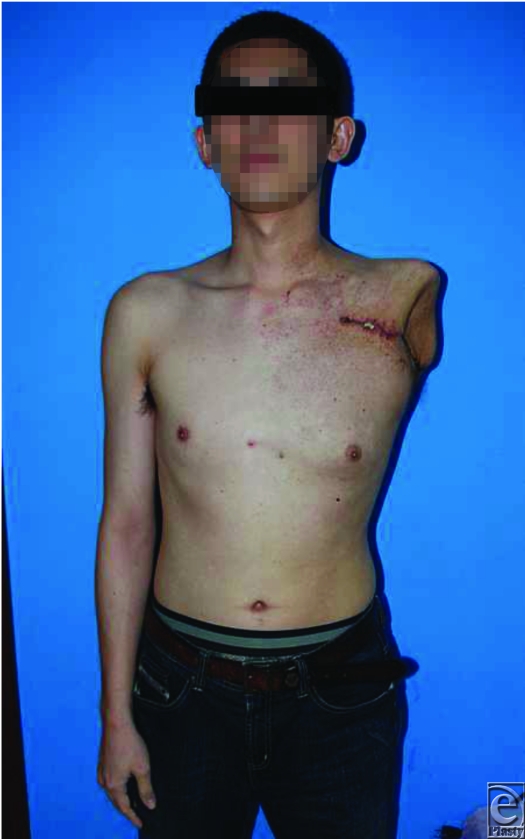
Uncomplicated recovery and normal wound healing.

A fasciocutaneous flap overlying the deltoid was raised and used for wound coverage. A suction drain was inserted and a soft, bulky dressing applied. Postoperative recovery, including wound healing, was unremarkable (Fig 4). The patient was discharged 2 weeks later. Cardiac failure soon resolved. One-year postsurgery, the patient remains well and has not encountered any wound problems.

## DISCUSSION

The management of AVM remains a significant challenge, particularly those of the upper limb. Intervention should only be contemplated when the lesion becomes symptomatic. A variety of treatment strategies have been reported with varying success.^2^^-^5

We believe that plastic surgeons should be an essential member of the multidisciplinary team treating such lesions. In some instances, surgical resection of the AVM with maximal preservation of upper-limb function may be required.

Clinical presentation of upper-limb AVMs can be diverse. Patients may suffer pain, nerve compression, ulceration, hand dysfunction, and spontaneous bleeding.^3^,^6^,^7^ Shunting through proximal arteriovenous fistulae may result in distal steal phenomenon manifesting as severe pain, ischaemia, and discoloration of the digits.^5^,^9^

Rarely, extensive arteriovenous shunting may lead to cardiac decompensation.^8^ This is an absolute indication for surgery. The presence of an upper-limb AVM does not correlate with vascular anomalies of other organ systems, nor does the pattern predict skeletal overgrowth.^5^,^9^

Management of AVM varies, with a conservative approach adopted for patients that are asymptomatic or have minor symptoms. If treatment is required, techniques that may be used include catheter embolization or direct percutaneous sclerotherapy. Embolization and sclerotherapy may be performed even after major surgery.

Surgical ligation of the main feeding vessels to the AVM often yields a poor result, as a collateral circulation subsequently develops.^10^ In our patient, surgical ligation of the subclavian vessels was performed as part of the second stage. This measure was undertaken to prevent uncontrollable bleeding during the shoulder disarticulation.

With the advent of highly selective embolization techniques, a number of authors have reported safe and successful outcomes when treating AVM with this modality.^6^ Indeed, it has been advocated as the treatment of choice by some.^8^

Regardless of the embolic agent used, the underlying aim is to obliterate the nidus. However, embolotherapy must not be considered a definitive cure and can unintentionally worsen symptoms. Furthermore, repeat embolization may be necessary to deal with recurrences.

In our patient, embolotherapy would not have been appropriate. The diffuse nature of the AVM with multiple nidi wound render embolotherapy ineffective. Our patient had a life-threatening, symptomatic, nonfunctional upper-limb AVM. In this situation, we believe that amputation or disarticulation should be explained to the patient at the outset. Amputation should be viewed as the “reconstructive” first step in the patient's rehabilitation.

We favored glenohumeral disarticulation rather than amputation through the neck of the humerus for a number of reasons. First, the latter provides no functional advantage, as the patient cannot grip or stabilize large objects between the stump and thorax. Second, even though the MRI indicated no apparent involvement of the humerus, amputation through the bone may result in significant bleeding. Third, the patient was not keen for preservation of proximal humerus with associated soft tissue reconstruction. His concern was a protracted postoperative stay and rehabilitation. The cosmetic deformity with glenohumeral disarticulation, however, is greater. Shoulder width and axillary contour are both lost.

Our rationale for a 2-stage amputation was based on the following: the aim of the first stage was to control the risk of bleeding and infection at the hand. Elbow disarticulation could be easily performed as a tourniquet could be applied to the arm. After control of infection, the second stage could be performed with the reduced risk of wound infection and breakdown.

In one series of 33 patients with high-flow AVM of the upper limb, the majority of patients were treated by surgical excision.^5^ However, in patients with diffuse lesions, 9 of 10 patients required major amputation despite repeated excision and reconstruction. White et al reported similar results, with 2 of 4 patients with high-flow diffuse AVM of the entire upper limb requiring amputation.^4^ Dickey et al also reported a 28% complication rate in all patients with high-flow lesions that underwent treatment.

A multidisciplinary approach is central to the effective management of AVMs. These lesions are complex and difficult to treat. Despite advances in embolic agents and microcatheter techniques, outcomes for high-flow upper-limb AVM is far from satisfactory. For diffuse lesions that are life-threatening, amputation as the first step in the patient's rehabilitation may be appropriate. Indeed, it has been previously stated that amputation was unnecessarily delayed in such patients.^5^

Surgical excision must be performed in a systematic manner, involving steps to minimize the risk of uncontrollable intraoperative bleeding and postoperative complications. In this case, successful amputation was achieved using temporary transcutaneous sutures to control bleeding from skin edges, ligation of the subclavian vessels, and glenohumeral disarticulation.
